# Experimental Realization of On‐Chip Surface Acoustic Wave Metasurfaces at Sub‐GHz

**DOI:** 10.1002/advs.202411825

**Published:** 2025-01-31

**Authors:** Wan Wang, Maciej Baranski, Yabin Jin, Roland Salut, Djaffar Belharet, Jean‐Michel Friedt, Yongdong Pan, Yanxun Xiang, Fu‐zhen Xuan, Abdelkrim Khelif, Sarah Benchabane

**Affiliations:** ^1^ School of Aerospace Engineering and Applied Mechanics Tongji University Shanghai 200092 China; ^2^ CNRS, FEMTO‐ST Université de Franche‐Comté 15B avenue des Montboucons Besançon F‐25000 France; ^3^ Shanghai Key Laboratory of Intelligent Sensing and Detection Technology School of Mechanical and Power Engineering East China University of Science and Technology Shanghai 200237 China; ^4^ Shanghai Institute of Aircraft Mechanics and Control Shanghai 200092 China; ^5^ College of Science and Engineering Hamad Bin Khalifa University Doha Qatar

**Keywords:** microfabrication process, on chip metasurface, scattered wave, subwavelength focusing, surface acoustic waves

## Abstract

Metasurfaces, consisting of subwavelength‐thickness units with different wave responses, provide an innovative possible method to manipulate elastic and acoustic waves efficiently. The application of metasurfaces to manipulate on‐chip surface acoustic wave (SAW) at sub‐GHz frequencies requires further exploration since their wave functions are highly demanded in nanoelectromechanical systems (NEMS), sensing, communications, microfluid control and quantum processing. Here, the experimental realization of on‐chip SAW metasurfaces is reported, consisting of gradient submicron niobium (Nb) rectangular pillars positioned on a 128°Y‐cut lithium niobate (LiNbO_3_) substrate that operate at hundreds of megahertz. The proposed SAW metasurfaces are able to manipulate transmitted SAW wavefront functions by designing on‐demand pillar's profile distributions. Broadband subwavelength focusing effects as the typical functions of SAW metasurfaces are experimentally demonstrated. This study opens a door for realizing on‐chip SAW metasurfaces for diverse potential applications at micro‐ and nanoscale.

## Graphical abstract

On‐chip surface acoustic wave (SAW) metasurfaces, composed of submicron niobium pillars on a lithium niobate substrate, are demonstrated to manipulate SAW wavefronts at hundreds of megahertz. With subwavelength total thickness and passive properties, these metasurfaces hold promise as integrated mechanical components for future acoustic quantum chips.

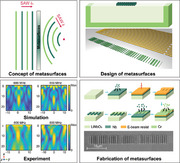

## 1 Introduction

**Figure 1:**
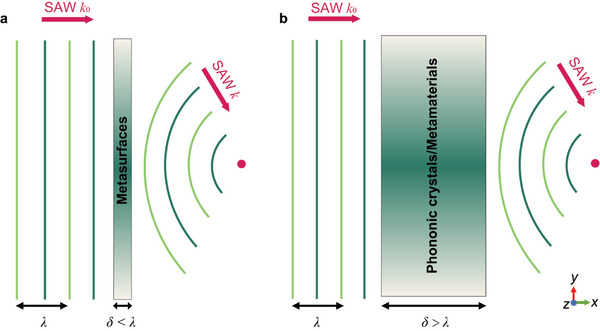
Illustration of a) metasurfaces and b) phononic crystals/metamaterials. *δ* represents the total thickness of metasurface or phononic crystals/metamaterials, while *λ* denotes the SAW wavelength.

The concept of metasurfaces, as illustrated in **Figure** **1**a, comprising subwavelength‐thickness units with nonhomogeneous phase response, offers a novel approach to efficiently manipulate wave phenomena. Compared with metamaterials^[^ ^1^, ^2^ ^]^ and phononic crystals,^[^ ^3^, ^4^ ^]^ whose overall thickness typically exceeds several wavelengths (Figure  1b), metasurfaces offer significant advantages due to their compact size, aligning with the highly integrated nature of modern chips and the imperative to mitigate power cost and minimize cross‐talk. Recently, metasurfaces have rapidly evolved in optical waves,^[^ ^5^, ^6^, ^7^ ^]^ microwaves,^[^ ^8^, ^9^ ^]^ acoustic waves,^[^ ^10^, ^11^, ^12^ ^]^ and extended to mechanical system,^[^ ^13^ ^]^ with numerous intriguing functionalities proposed, such as focusing,^[^ ^14^, ^15^, ^16^ ^]^ source illusion,^[^ ^17^ ^]^ or total internal reflection.^[^ ^18^ ^]^ Various designs have been proposed to control the elastic waves, including space curl structure,^[^ ^17^ ^]^ tailored structure^[^ ^19^ ^]^ and resonant pillar.^[^ ^20^ ^]^ Most research focused on macro‐scale structures and plate‐type or bulk media, however, their complex structures make it difficult to be applied to submicron or nanoscale. This limitation hinders the practical application of elastic metasurfaces particularly in quantum chips and communication science.^[^ ^21^ ^]^

Surface acoustic waves (SAW), serving as an excellent platform for working with high‐frequency signals, have garnered significant attention since the last century. SAW devices are widely utilized in current radio frequency (RF) industries, such as filter, delay line, resonators and microfluid chips. In recent years, numerous new promising applications of SAW have been proposed, spanning classical sensing,^[^ ^22^, ^23^ ^]^ actuation,^[^ ^24^, ^25^ ^]^ and quantum science,^[^ ^26^, ^27^ ^]^ highlighting an urgent demand for arbitrary design of SAW wavefronts at micro‐nano scale. The control of chip‐scale SAW has typically been achieved using electrical electrodes. Due to their advantages in ease of manufacturing through microfabrication, interdigital transducers (IDTs) consist of arrays of metallic electrodes are commonly used not only to excite plane SAW,^[^ ^28^ ^]^ but also to obtain focused SAW.^[^ ^29^, ^30^ ^]^ However, given the active properties and electromechanical coupling mechanisms, the focused IDTs limits the efficient SAW generations and broad‐field focusing within the chip. The dislocated interdigital transducer,^[^ ^31^ ^]^ as a type of phased array active structure, offers a better solution to mitigate the electromechanical coupling efficiency compared to the focused IDT. While electrode generators offer real‐time tunability and broad control flexibility, mechanical and passive components provide different advantages for within‐chip control, especially in enabling complex out‐of‐source modulation.

Besides the active solutions mentioned above, SAW modulation can be effectively achieved using artificially structured materials like phononic crystals and metamaterials. By incorporating line defects, phononic crystals enable micro‐scale SAW waveguides with functionalities such as splitting and bending,^[^ ^32^ ^]^ while the integration of topological concepts allows for the design of robust waveguides.^[^ ^33^ ^]^ Acoustic lenses can be realized either through the anisotropic propagation characteristics of tungsten/lithium niobate phononic crystals^[^ ^34^ ^]^ or by employing gradient‐index phononic crystal.^[^ ^35^, ^36^ ^]^ Moreover, metamaterials provide a means to achieve SAW attenuation,^[^ ^37^, ^38^ ^]^ extending the range of functionalities offered by structured materials. Although phononic crystals/metamaterials offer diverse functionalities, subwavelength metasurfaces present a superior choice for controlling SAW as menthioned at the begining. Screw‐nut structures are also employed for wavefront modulation of Rayleigh waves ≈40 kHz.^[^ ^39^ ^]^ Sub‐wavelength resonant mass‐spring microstructures are used to achieve local acoustic field gradients at 7 MHz.^[^ ^40^ ^]^ Sawtooth‐based metasurfaces leveraging diffractive effects are proposed for flexibly steering acoustic fields in microfluidic device ≈40 MHz.^[^ ^41^ ^]^ Such subwavelength metassurfaces provide a new efficient way in SAW microfluidics.^[^ ^42^ ^]^ However, how to achieve SAW subwavelength metasurfaces at higher frequencies such as sub‐giga Hertz remains challenging.

To address the aforementioned challenges, we propose a new approach to realize on‐chip SAW metasurface. Due to the significant advantages of the pillar‐based platform^[^ ^43^, ^44^ ^]^ in microfabrication, we design the metasurface unit consisting of a series of identical pillars to enhance their resonant modes that can generate efficient phase shift and high amplitude for transmitted SAWs. We further investigate mechanisms for efficient phase modulation by analyzing the scattering property of pillar's resonant modes and the interference between the scattered and incident SAWs. By designing varied width and thickness of rectangular pillars in metasurface units, the on‐chip SAW metasurfaces are able to manipulate wavefront shaping of the transmitted SAWs. We present the design methodology for broadband subwavelength focusing effect as an example of wave functions.^[^ ^45^, ^46^ ^]^ A microfabrication process including E‐beam lithography and plasma etching is utilized for sample preparation. The capability for broadband focusing is emphasized through both simulations and experiments to validate the proposed on‐chip SAW metasurfaces that have potential applications at micro‐ and nanoscale for NEMS, sensing, communications, quantum processing, among others.

## 2 Metasurface Units Design

**Figure 2:**
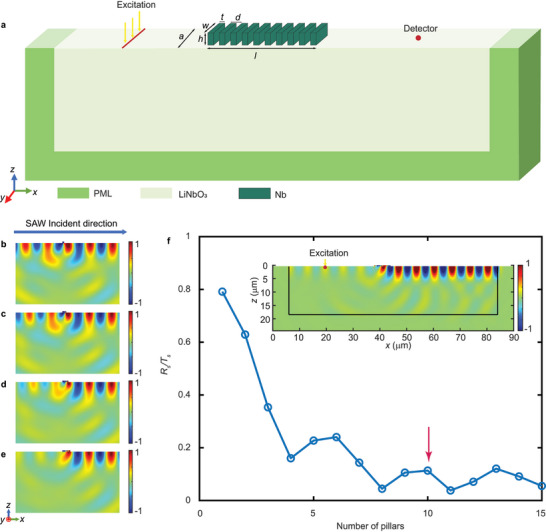
a) Illustration of the metasurface units. (b)–(e) shows the scattering fields of metasurface units with 1 to 4 pillars, respectively. f) variation of *R_s_*/*T_s_* as a function of the number of pillars, where *R_s_* represents the amplitude of reflected scattering wave and *T_s_* represents the amplitude of transmitted scattering wave; Inset is the scattering field with 10 pillars.

The key in designing an efficient metasurface lies in obtaining a 2π span of the transmitted phase alongside high amplitude. Unlike Lamb waves, introducing a single resonating pillar acting as a point source for SAW modulation proves challenging due to the radiation into the bulk of the piezoelectric substrate. In the study of on‐chip SAW metasurface units, we investigate the response of a group of resonating pillars under SAW excitation. The SAW metasurface unit consists of several identical niobium (Nb) rectangular pillars positioned on a 128°Y‐cut lithium niobate (LiNbO_3_) substrate, as illustrated in **Figure**  **2**a. The Young's modulus of niobium is 105.3 GPa, with a Poisson's ratio of 0.39 and a density of 8571.3 kg m^−^^3^. The continuity condition is applied to both sides along the y‐axis direction to simulate an infinite structure and avoid boundary effects. Additionally, Perfectly Matched Layer (PML) surrounding the model is employed to minimize reflections from the boundaries. SAW is launched along *x* direction by vertical force excitation, and the transmitted waves are subsequently detected on the other side of substrate surface. More details about the numerical simulations can be found in Supporting Information.

The first step in unit design is to choose the optimal number of pillars. We set the lattice constant *a* = 1.5 µm, the height of pillars *h* = 0.45 µm, interval between two pillars *d* = 0.6 µm, thickness of pillar *t* = 0.5 µm, width of pillar *w* = 0.914 µm and working frequency *f* = 600 MHz, corresponding to a simulated wavelength *λ* = 6.7 µm. These geometrical parameters are obtained through a broad search and correspond to a specific resonance state, which will be described later. The resonant pillar acts as a secondary excitation source, stimulating the scattered wave; and the resulting transmitted wave emerges from the destructive interference between the incident wave and the scattered wave.^[^ ^47^ ^]^ To delve deeper into the working mechanisms of metasurface units, a scattering field analysis was conducted. The scattering field can be derived by subtracting the out‐of‐plane displacement field in the presence of the pillars from that without them.

In Figure  2b–e, we present the scattering fields for pillar numbers ranging from 1 to 4, respectively. It can be observed that when there is only a single bending‐resonance pillar, it behaves like a dipolar point source, with its transmitted and reflected scattered fields being of comparable strength, as shown in Figure  2b. As the number of pillars increases, their reflected scattering waves exhibit destructive interference, while the transmitted scattering waves demonstrate constructive interference. This can lead to a reduction of reflected energy and a corresponding increase in transmission efficiency. To make this change more intuitively demonstrable, we define a coefficient *R_s_/T_s_*, where *R_s_* represents the amplitude of reflected scattering wave and *T_s_* represents the amplitude of transmitted scattering wave. A ratio of 1 indicates a similar intensity of reflected and transmitted scattering, while a ratio close to 0 signifies almost no reflection, achieving full transmission. Figure  2f illustrates the relationship between *R_s_/T_s_* and the number of pillars. It is evident that further increasing the number of pillars results in negligible gains in transmittance efficiency while significantly enlarging the size, thereby diminishing the compactness advantage of metasurfaces. Considering the balance between efficiency and the overall dimensions of the structure, a 10‐pillar configuration is chosen as the metasurface unit, and its scattering field is shown in the inset of Figure  2f. With such geometric size and operating frequency, the total length *l* of the proposed metasurface is ≈6 µm, less than one wavelength. As a side note, the results for transmitted waves (a combination of incident and scattering waves) can be found in the Supporting Information.

**Figure 3:**
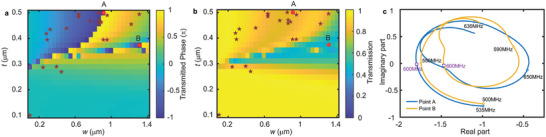
Variation of a) transmitted phase and b) transmission as a function of the thickness *t* and width *w* of pillars; red dots A and B represent two typical modes; red stars show the selected points for focusing metasurfaces. c) The Nyquist plot of scattering waves emitted by metasurfaces units at point A (blue curve) and B (yellow curve); The purple box represents the design frequency as 600MHz.

To achieve 2π phase variation, the thickness *t* and width *w* of pillars can be adjusted to introduce different resonance states of the unit. These parameters were primarily chosen to align with the 2D patterning preferences and precision limitations of standard microfabrication techniques. The transmitted phase and amplitude transmission are defined as the ratio of the out‐of‐plane displacement of the transmitted wave (with pillars) to that of the incident wave (without pillars). By varying the width (from 0.1 to 1.4 µm) and thickness (from 0.1 to 0.5 µm) of the pillars, **Figure**  **3**a, b depicts the transmitted phase and transmission coefficient of displacement as functions of pillar thickness and width, respectively. Two types of out‐of‐phase patterns with distinct transmission are observed at point A *t* = 0.5 µm, *w* = 0.914 µm and point B *t* = 0.374 µm, *w* = 1.311 µm, demonstrating high and low transmission efficiency, respectively. The geometrical parameters as point A are also used for the investigation of the pillar numbers. The two patterns occur very close to each other in the *w*‐*t* plane, making it impossible to achieve efficient phase modulation only considering one geometric parameter. For example, when 𝑡 = 0.4 and 𝑤>0.8, the pillar resonance state transitions into the low transmission resonance mode B before fully exiting resonance mode A. This results in a smaller transmission coefficient in the phase interval of [0, 0.5].

To elucidate the different modes observed at points A and B, denoted by red dots in Figure  3a,b, a frequency domain analysis ≈600 MHz was conducted. The transmitted scattering wave relative to the incident wave is defined as $w_{s}=\frac{w_{p}-w_{\mathrm{ref}}}{w_{\mathrm{ref}}}\;$, where *w*_p_ and *w*_ref_ are the out‐of‐plane displacement of the transmitted wave (with pillars) and the incident wave (without pillar) at detected point. In Figure  3c, The Nyquist plot of scattering wave *w*_s_ is displayed. The x‐axis represents the real part of *w*_**s**_, while the y‐axis represents the imaginary part. Each point on the curves corresponds to the scattering response at a specific frequency. The distance from a point to the origin indicates the amplitude of the scattering wave relative to the incident wave, and the angle between the line connecting the point to the origin and the x‐axis represents the phase of the scattering wave relative to the incident wave. The metasurface units at points A (blue curve) and B (yellow curve) exhibit similar responses, depicting a spiral shape that contracts toward the center as the frequency increases, demonstrating the diversity of resonance modes. However, at the operating frequency of 600 MHz (purple box), the two points are in different resonance modes. In the Nyquist plot, the imaginary parts of the two points are ≈0, and their real parts are negative, indicating that the scattered waves at both points are out‐of‐phase with respect to the incident wave. Specifically, the real parts of points A and B are ≈−1.8 and −1.47, which means that the amplitude of the out‐of‐phase scattered waves at the two points are 1.8 and 1.47 times of that of the incident wave, respectively. Considering destructive interference between the incident and scattering waves, the transmission is 0.8 and 0.47 for point A and B respectively, meanwhile both of them are out‐of‐phase. The geometric parameters with high transmission like point A will be selected for on‐chip SAW metasurface design.

## 3 On‐Chip SAW Metasurface Design

**Figure 4:**
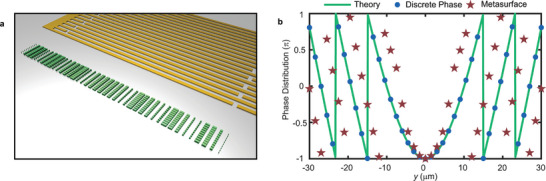
a) Demonstration of focusing model; green blocks are the on‐chip SAW metasurface and yellow part denotes the IDT used for excitation; b) continuous phase profile (green line) for *F* = 2λ, discrete phases based on the position of each unit (blue dots) and final phase of metasurface units (red stars).

The objective of this study is to design metasurfaces composed of distinct pillars capable of transforming an incident wave into desired wavefront patterns, including but not limited to splitting, beam deflection, focusing, and source illusion. Hereby, we concentrate on showcasing the focusing capability of the proposed metasurfaces, a functionality commonly employed for energy concentration and sensing. The plane wave focusing model can be designed based on the generalized Snell's law as demonstrated in **Figure**  **4**a. The metasurface comprises 41 units (marked as green), with the pillars in each unit varying in thickness and width to achieve different phase responses. A chirped (IDT) is used to launch SAW in the experiment. To determine the phase of each unit, we have to start from the continuous phase response profile φ(*y*) along the metasurface, which can be given by $\;\varphi \left(y\right)=\frac{2\pi }{\lambda }\;\left(\sqrt{F^{2}+y^{2}}-F\right)$ where *λ* = 6.7 µm is the working wavelength, *F* is the focal length and *y* is the y‐coordinate position along the metasurface. Since the metasurface is constructed from individual pillared units that cannot characterize continuous phase responses, it becomes necessary to discretize the continuous phase based on the position of the units along the metasurface. Furthermore, given the anisotropic properties of the LiNbO_3_ substrate, phase compensation caused by material is essential, operating on a discrete phase basis. Phase compensation of *i*th unit can be written as $\;\psi_{i}\;\left(y_{i}\right)=\frac{2\pi (\lambda -\lambda_{i})}{\lambda \lambda_{i}}\;\sqrt{F^{2}+{y_{i}}^{2}}$ where λ_*i*_ represents the wavelength of the SAW propagating at an angle of arctan(*y_i_*/*F*) degrees to the *x*‐coordinate. More details about phase compensation can be found in Supporting Information. In Figure  4b, the continuous phase profile (green line) for *F* = 2λ focusing is calculated and 41 discrete phase points (blue dots) are selected according to the unit positions. The choice of a small focal length is intended to better demonstrate the sub‐wavelength focusing effect.^[^ ^20^ ^]^ The final phases utilized for the metasurface units, after incorporating material phase compensation with discrete phases, is represented by the red stars. The significant discrepancy between the discrete phases and the metasurface phases underscores the importance of accounting for the effects of anisotropic materials.

Once the phase of metasurface units and the relationships between transmitted wave and geometric parameters of pillars are determined, the final step is to identify the optimal thickness and width of the pillars. Since we sweep both thickness and width, structures with the same phase can correspond to various geometric parameters. To achieve maximum efficiency, a fundamental principle involves selecting points with the highest transmission coefficients in Figure  3a,b that correspond to the target phase. The selected points are shown as red stars in Figure  3a,b, with an average transmission of 0.85, indicating sufficient energy in the focusing field. All the geometrical parameters of focusing metasurface can be found in Supporting Information.

## 4 Device Fabrication

**Figure 5:**
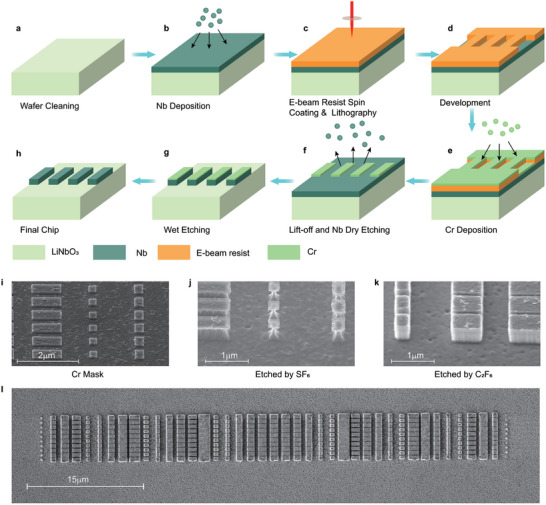
a–h) Illustration of the pillar fabrication process. i) Cr mask for plasma etching. j) SF_6_ and k) C_2_F_6_ Etched pillars. l), top view of the focusing metasurface.

The fabrication of the entire device can be divided into two stages: the preparation of the metasurfaces and the preparation of the IDT. Due to the material properties of niobium, combined with the hundred‐nanometer structural scale and the high aspect ratio of the pillars, the fabrication of the on‐chip SAW metasurface presents significant challenges. **Figure**  **5**a–h illustrates our fabrication process of the Nb pillars. Initially, a 450 nm Nb layer was deposited onto the cleaned wafer surface using sputtering with Plassys MP500. Patterns were then created using PMMA 672.03 resist through E‐beam lithography (Raith Voyager). Subsequently, an evaporation of Cr for the plasma etching mask was carried out using Plassys MEB600, followed by a lift‐off process to obtain the final mask. Plasma etching was conducted using Corial 200R to form the corresponding Nb pillars. Lastly, wet etching was employed to remove residual masks from the plasma etching process. The fabrication of the IDT follows a more standard process compared to the preparation of the Nb pillars. We utilized E‐beam lithography with CSAR 18 resist to create the pattern, followed by the deposition of 100 nm Al electrodes through evaporation and lift‐off steps. Chirped IDT properties can be found in Supplementary materials.

There are two key considerations during the fabrication process: 1) selecting the appropriate mask for plasma etching, and 2) configuration of the etching gases to achieve a vertical profile of the pillars. Due to the structures in the hundreds of nanometers range, high‐precision E‐beam lithography is essential, which limits the thickness of the deposition layers. Therefore, the material must be sufficiently hard during dry etching to ensure the mask can be as thin as possible. Additionally, the mask should be easily removable using an Nb‐insensitive wet etching solution. The Cr mask, as a plasma etching mask, perfectly meets the aforementioned conditions, as shown in Figure  5i. The thickness of the Cr mask is only 65 nm, which is only 14.4% of the pillar's height. Once the mask is determined, the attempt of plasma etching can be performed. Figure  5j,k shows results of plasma etching using two based on different plasma gases: 25 sccm SF_6_ (7 mTorr, 70 Watt) and 25 sccm C_2_F_6_ (with 5 sccm O_2_ and 2 sccm Ar to increase the etch rate, 15 mTorr, 90Watt). In case of SF_6_‐based plasma etching (Figure  5j) large under‐etching in lateral direction occurred, possibly due to partial chemical etching of SF_6_ plasma. Conversely, Figure  5k displays a structure with desired vertical side walls, leading us to use C_2_F_6_ for the fabrication of devices. The final metasurface with 449 nm height is shown in Figure  5l.

## 5 Results

**Figure 6:**
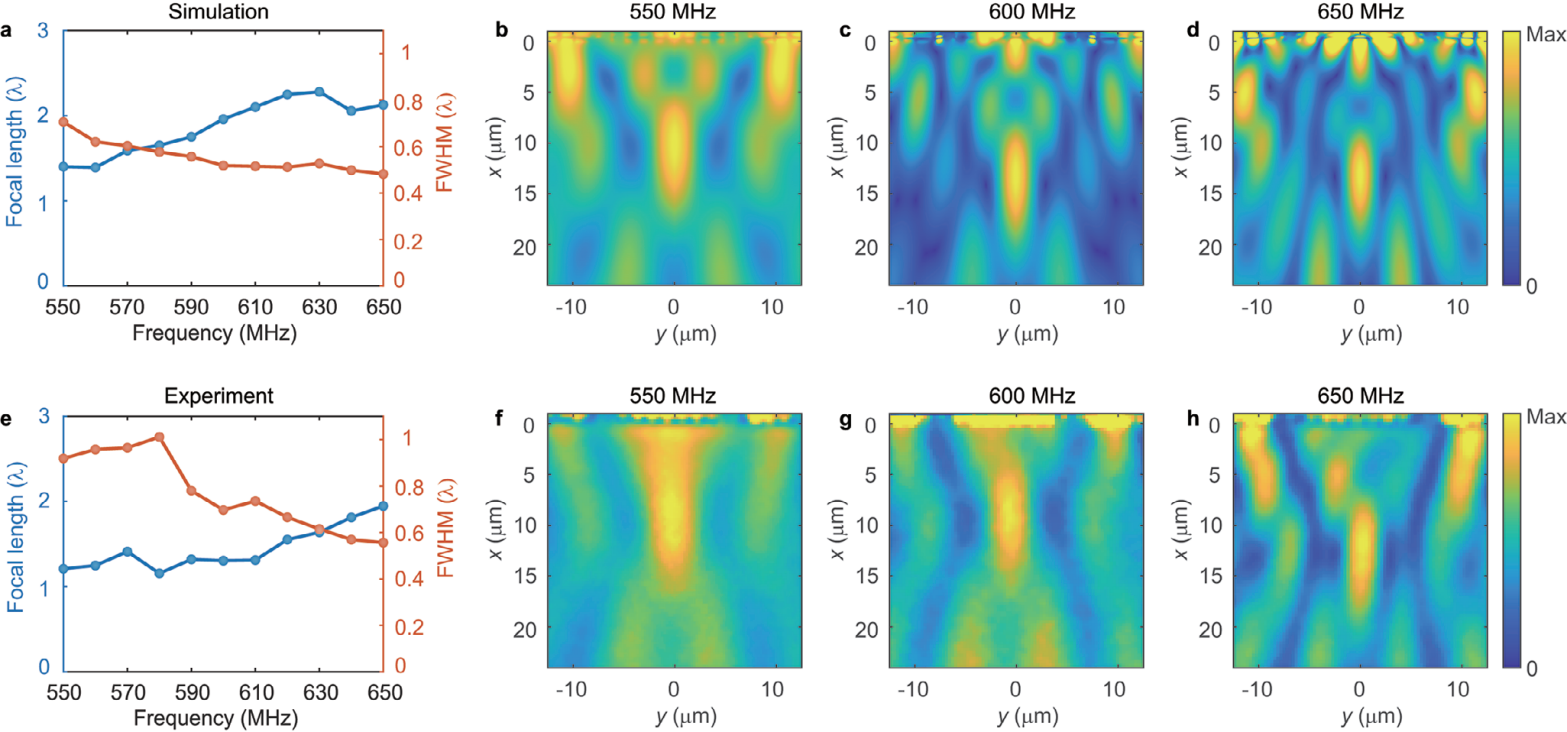
Variation of focal length (blue curve) and FWHM (red curve) along y‐axis as a function of frequency in a) simulation and e) experiments. The amplitude fields at frequencies of b,f) 550 MHz, c,g) 600 MHz, and d,h) 650 MHz. The first row displays the simulation results, while the second row presents the experimental findings.

In the experiments, RF signals with a power level of 15 dBm are applied to the interdigital transducer to generate the surface acoustic wave. Subsequently, a heterodyne interferometric optical probe is used to scan the 25 µm × 25 µm focusing area located in front of the midpoint of the metasurface. The optical probe, out‐of‐plane acoustic field mapping measurement setup, is described in detail in the Supporting Information. At our design frequency of 600 MHz, the measured wavelength is 6.57 µm, which deviates by only 2% from the simulated wavelength. Despite this minor difference, simulated wavelength is utilized in all subsequent analyses. **Figure**  **6**c,g displays the amplitude fields from both simulation and experiment. Clearly discernible focusing spots in these figures provide strong evidence supporting the focusing capability of the SAW metasurface. The simulated focal length is 1.96λ, closely aligning with our designed focal length of 2λ as discussed in the metasurface design section and the experimentally measured focal length is 1.31λ. We attribute this discrepancy to several factors including variations in the material properties of niobium due to deposition conditions, imperfections in the shape of the pillars, and other factors potentially influencing the resonance states.

We also examined the broadband properties of these metasurfaces across a frequency range from 550 to 650 MHz, achieving a frequency bandwidth ratio (bandwidth divided by the central frequency) of ≈16.7%. For a more detailed comparison, we analyzed the size of the focal spot and focal length relative to the corresponding wavelength as a function of frequency. These comparisons are illustrated in upper (simulation) and lower (experiments) panels of Figure  6. Both the simulated and experimental focal lengths exhibit a slight increase with rising frequency, while the full width at half maximum (FWHM) along the *y*‐direction gradually decreases, indicating a consistent trend between the experimental and simulated results. For a more visual comparison, the amplitude fields at frequencies of 550, 600, and 650 MHz are presented separately for both simulation and experiment in Figure  6b–d,f–h. Notably, the maximum value indicated on the colorbar corresponds to the peak amplitude at the respective focal point. It is observed that the FWHM of the focal spot remains subwavelength in a broad band that is a remarkable feature of the on‐chip SAW metasurface.

## 6 Discussion

**Figure 7:**
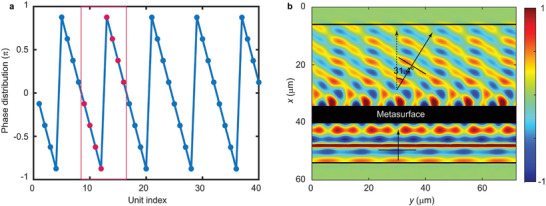
a) Phase distribution along the metasurface, with red dots indicating the phases of subunits within a supercell. b) Real part of the out‐of‐plane displacement field illustrating the deflection.

The overall results observed from this work suggest that a good focusing effect with broadband robustness can be achieved using different resonance modes of multiple submicron pillars. Notably, in addition to the focusing phenomenon, other wavefront modulations such as splitting and deflection can be easily achieved by employing different phase distribution functions. Here, we demonstrate a deflection case as an example. For a beam of normal incident SAW, achieving a deflection at an angle 𝜃 requires a phase distribution along metasurface given by $\varphi \;\left(y_{i}\right)=\frac{2\pi }{\lambda (\theta )}\;sin\left(\theta \right)y_{i}$ where λ(θ) is the new wavelength after deflection, and *y_i_* denotes the position of the 𝑖th unit. The design angle 29.1° is chosen because the unit phases along the metasurface exhibit a periodic distribution, as shown in **Figure**  **7**a. Consequently, by applying periodic boundary conditions, computational resources are optimized by focusing on a supercell comprising eight subunits. The phases of these eight subunits are indicated as red dots in Figure  7a. In Figure  7b, the real part of the out‐of‐plane displacement is presented, clearly demonstrating a deflection effect of ≈31.4°.

Theoretically, this work extends the concept of elastic metasurfaces from the macroscopic scale to the submicron scale. The positive results obtained both theoretically and experimentally demonstrate the feasibility of the on‐chip SAW metasurfaces for micro‐nano scale chip and device applications. In terms of applications, specifically SAW manipulation, such metasurfaces offer advantages of simple structure and overall subwavelength size compared to conventional focused interdigital transducers or phononic crystals with periodic structures. Due to their compactness and passivity, these metasurfaces can be used in the future for directional manipulation of acoustic energy within quantum acoustic chips.^[^ ^21^ ^]^

## 7 Conclusion

In summary, we introduced theoretical and experimental realization of on‐chip SAW metasurfaces composed of multiple pillared units designed for SAW manipulation. By analyzing the effects of scattered waves reemitted by the resonance modes of the units, we demonstrated that a 2π phase span with high transmission coefficients can be achieved by adjusting the width and thickness of the pillars. We illustrate this capability through the design of the SAW metasurface and show a microfabrication process for device preparation. Chromium is utilized as the etching mask, while C_2_F_6_ serves as an effective etching gas for niobium. The amplitude fields obtained from optical probe and simulations exhibit a strong agreement in trends, underscoring the broadband properties of such metasurfaces. Furthermore, these metasurfaces at micro‐nanoscale can be adapted for various functions, including deflecting waves and splitting, offering significant potential for applications in microelectromechanical systems, sensing, electronic chips, quantum devices, and beyond.

## Conflict of Interest

The authors declare no conflict of interest.

## Supporting information



Supporting Information

## Data Availability

The data that support the findings of this study are available in the supplementary material of this article.
