# Supplementary material for: Experimental Realization of On‐Chip Surface Acoustic Wave Metasurfaces at Sub‐GHz
Source: Adv Sci (Weinh). 2025 Jan 31;12(12):2411825. doi: 10.1002/advs.202411825 (PMC11947993; doi:10.1002/advs.202411825)
Supplement: Supplementary file 1 — Supporting Information [file ADVS-12-2411825-s001.docx]

**Supplementary materials**

**Experimental realization of on-chip surface acoustic wave metasurfaces at sub-GHz**

Wan Wang^1,2^, Maciej Baranski^2^,Yabin Jin^3,4,*^, Roland Salut^2^, Djaffar Belharet^2^, Jean-Michel Friedt^2^, Yongdong Pan^1^, Yanxun Xiang^3^, Fu-zhen Xuan^3^, Abdelkrim Khelif^2,5^, Sarah Benchabane^2,*^

^1^School of Aerospace Engineering and Applied Mechanics, Tongji University, 200092 Shanghai, China

^2^Université de Franche-Comté, CNRS, FEMTO-ST, 15B avenue des Montboucons F-25000 Besançon, France

^3^Shanghai Key Laboratory of Intelligent Sensing and Detection Technology, School of Mechanical and Power Engineering, East China University of Science and Technology, 200237 Shanghai, China

^4^Shanghai Institute of Aircraft Mechanics and Control, 200092 Shanghai, China

^5^College of Science and Engineering, Hamad Bin Khalifa University, Doha, Qatar

^*^Corresponding author: yabin.jin@ecust.edu.cn; sarah.benchabane@femto-st.fr

**1. Numerical simulations**.

All simulations were conducted using the Finite Element Method through frequency domain calculations in COMSOL. Considering the piezoelectric effect of lithium niobate, we employed the Solid Mechanics and Electrostatics modules for substrate calculations, while the niobium pillars were solely analyzed using the Solid Mechanics module. An out-of-plane line force was applied to the substrate surface to excite surface acoustic waves. For unit simulations, perfectly matched layers (PML) are positioned at the wave propagation end and the substrate bottom to minimize wave reflection. Periodic boundary conditions were implemented on the substrate side perpendicular to the wave propagation direction to simulate infinite units. For the focusing model, PML are employed to minimize boundary effects, while symmetry conditions are applied to reduce computational time and resources.

**2. Metasurface units for different numbers of pillars.**

The primary consideration in selecting the number of pillars is the transmission efficiency while achieving out-of-phase with respect to the incident wave. Fig. S1 illustrates the variation of the transmitted phase and transmission as a function of the number of pillars. The calculations for this figure were based on the same geometrical parameters as point A in Fig.3a. It is worth noting that structures with fewer than 10 pillars are not in the strongest resonance state (i.e., have not achieved out-of-phase property). Consequently, their transmission, when attempting to realize out-of-phase, will be significantly weaker than in the current configuration. This result indicates that with around 10 pillars, the highest transmission can be achieved, while also realizing the out-of-phase phenomenon. Another secondary consideration is minimizing the overall size of the metasurface to fully leverage its subwavelength compactness. Considering all factors, the gain in transmittance efficiency from further increasing the number of pillars becomes negligible, which is why values exceeding 10 such as 11 were not considered.


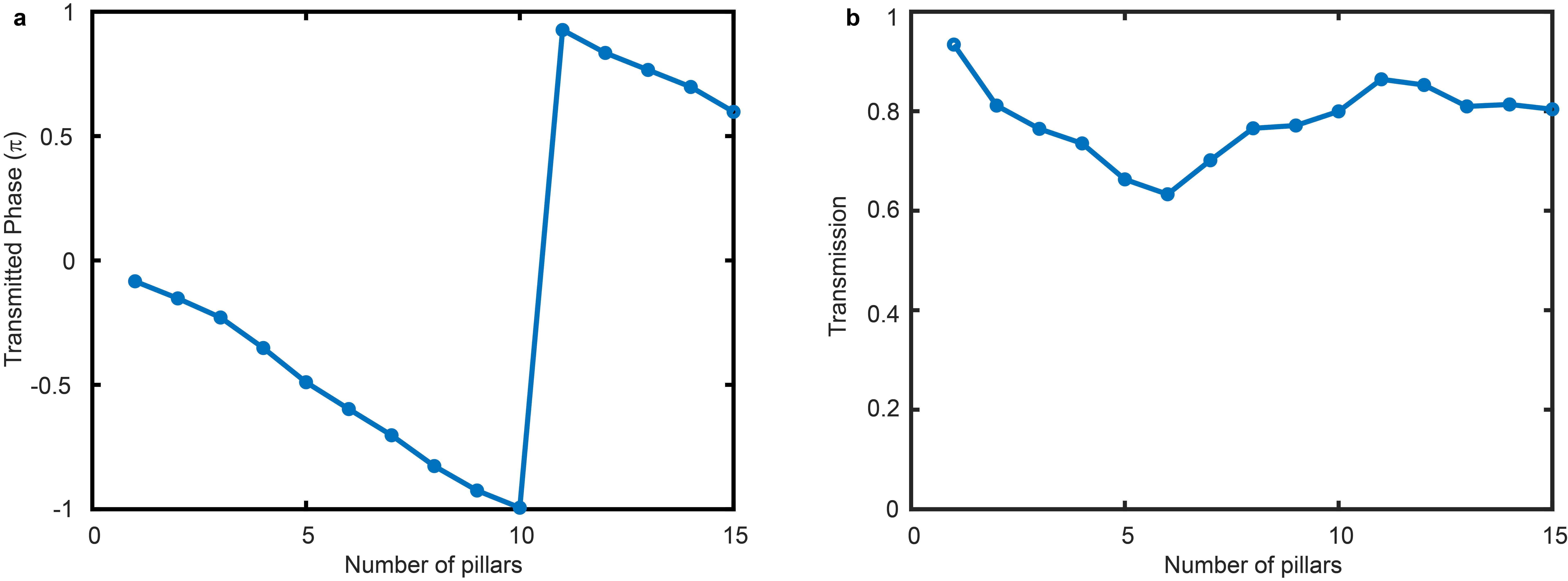


Fig.S1 Variation of (a) transmitted phase and (b) transmission as a function of the number of pillars with the same geometrical parameters as point A in Fig.3a.

**3. Phase compensation caused by** **anisotropic material properties**

As an anisotropic material, lithium niobate exhibits distinct surface acoustic wave velocities in different directions. In the context of plane wave focusing, the variation in refractive index causes a shift in the propagation direction of the plane wave as it passes through the metasurface, resulting in a change in the wave velocity that must be carefully considered. Since the wavelength can be directly related to the wave velocity, we present the FEM model used to calculate the wavelength at 600 MHz in different directions in Fig.S2. Due to the symmetry of the material properties of lithium niobate, a quarter of the cylinder is used for wavelength calculations to conserve computational resources. Perfectly matched layers are applied to the outer and bottom domains of the cylinder to minimize boundary reflections. Symmetry conditions are applied to the cut surface of the quarter-cylinder to simulate a full-angle scenario. A point source is positioned at the center of top surface of the cylinder to excite the circular SAWs. Two probe lines, line 1 and line 2, are placed at distances of 5λ_0_ and 7λ_0_ from the point source, where λ_0_ represents the wavelength of propagation along the x-direction at 600 MHz.


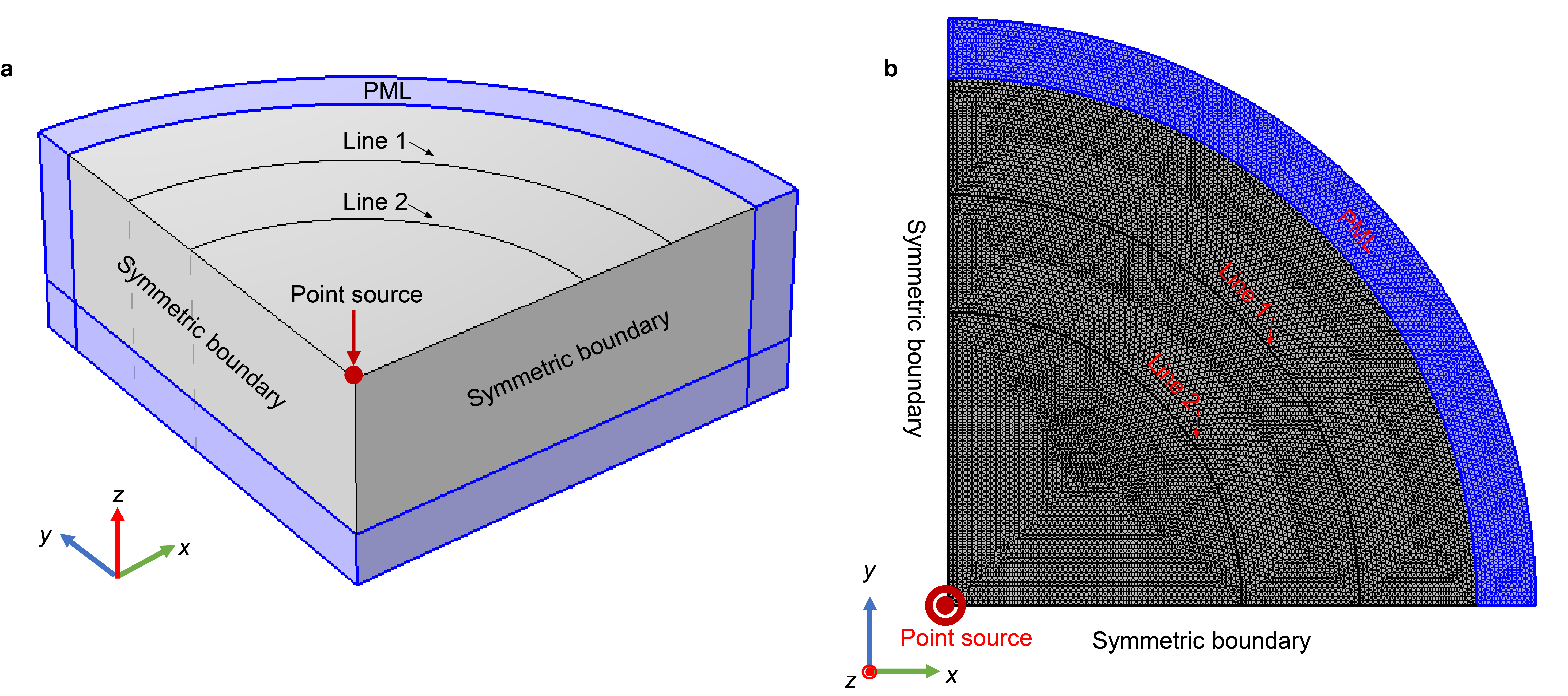


Fig.S2 (a) Axonometric view and (b) top views of the model used to calculate the wavelength of lithium niobate in different directions.

The out-of-plane displacements obtained at the two probe lines are denoted as *w*_1_ and *w*_2_ respectively. The unit phase difference between the two probe lines can be written as

$$\begin{aligned} \Phi\left( \theta\right)=arg\left( \frac{w_{2}\left( \theta\right)}{w_{1}\left( \theta\right)} \right)/2\lambda_{0}\#\left( 1 \right) \end{aligned}$$

where $\theta$ is the angle with the x-direction. Further, the wavelength can be written:

$$\begin{aligned} \lambda\left( \theta\right)=2\pi/\left( \Phi\left( \theta\right)+2\pi/\lambda_{0} \right)\#\left( 2 \right) \end{aligned}$$

The result of $\lambda(\theta)$ can be found in Fig.S3. Once the wavelengths at each angle are determined, the compensating phase can be calculated. The angle of *i*th unit of metasurfaces is

$$\begin{aligned} \theta_{i}=arctan\left( y_{i}/F \right)\#\left( 3 \right) \end{aligned}$$

where $y_{i}$ is the location of the unit, and *F* is the focal length. The distance between *i*th unit and the focal point is

$$\begin{aligned} L_{i}=\sqrt{{y_{i}}^{2}+F^{2}}\#\left( 4 \right) \end{aligned}$$

The phase compensation caused by unit distance can be written as

$$\begin{aligned} \eta_{i}=\frac{2\pi}{\lambda\left( \theta_{i} \right)}-\frac{2\pi}{\lambda_{0}}\#\left( 5 \right) \end{aligned}$$

Thus, phase compensation of *i*th unit of metasurfaces can be written as

$$\begin{aligned} \psi_{i}\left( y_{i} \right)=\eta_{i}L_{i}=\frac{2\pi\left( \lambda_{0}-\lambda\left( \theta_{i} \right) \right)}{\lambda_{0}\lambda\left( \theta_{i} \right)}\sqrt{F^{2}+{y_{i}}^{2}}\#\left( 6 \right) \end{aligned}$$

The final phases of metasurface units are

$$\begin{aligned} \varphi\left( y_{i} \right)=\frac{2\pi}{\lambda}\left( \sqrt{F^{2}+{y_{i}}^{2}}-F \right)-\psi_{i}\left( y_{i} \right)\#\left( 7 \right) \end{aligned}$$

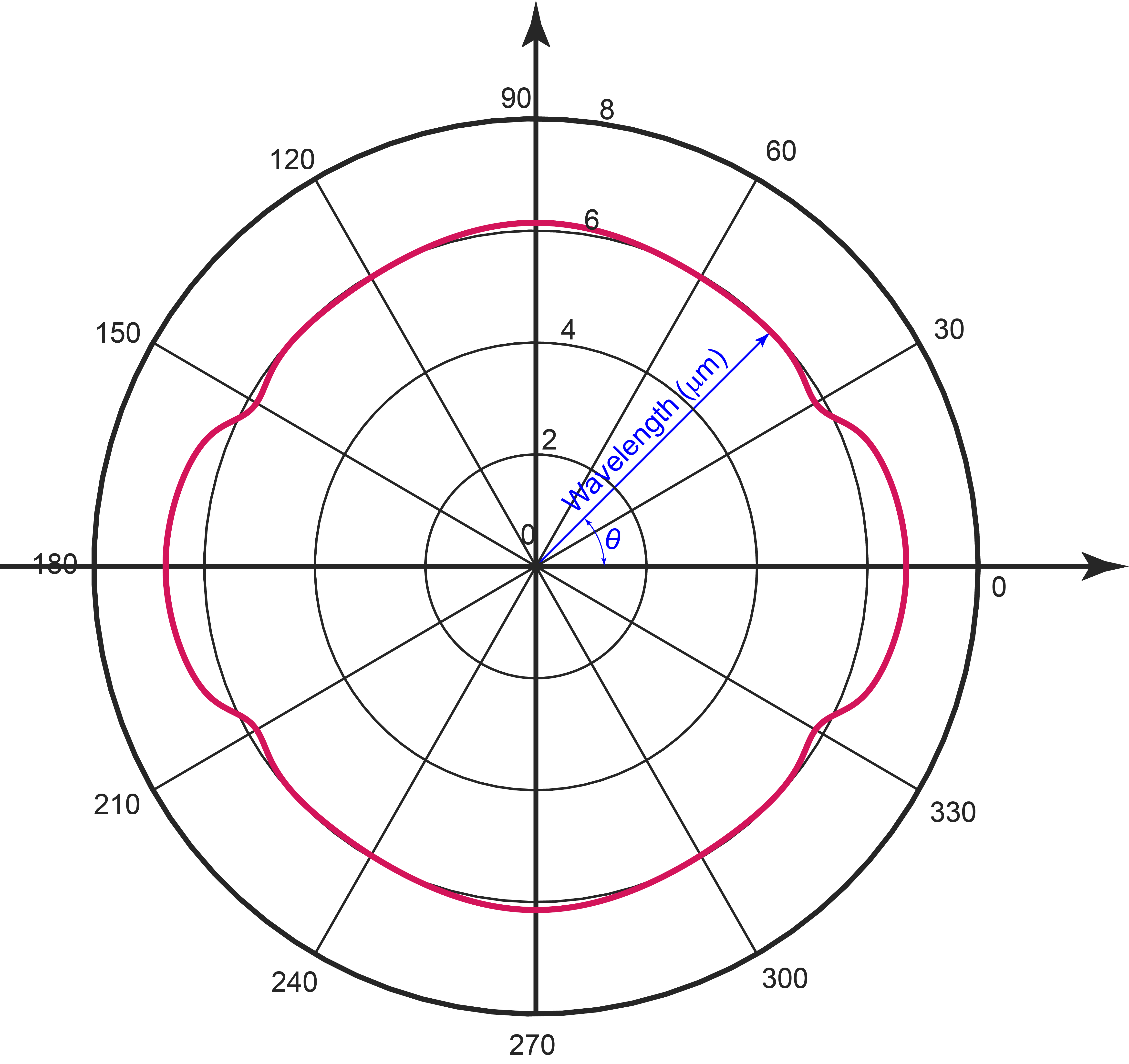


Fig.S3 Wavelength variation diagram of lithium niobate at different angles with respect to the x-direction.

**4. Geometrical parameters of focusing metasurface**

The specific geometric parameters of the metasurface used for focusing surface acoustic waves (SAWs), including thickness (𝑡) and width (𝑤), are listed below.

Table S1 Geometrical parameters of focusing metasurface

| *i-*th unit | *w* (µm) | *t* (µm) |
| --- | --- | --- |
| 1 | 0.100 | 0.100 |
| 2 | 0.504 | 0.439 |
| 3 | 0.852 | 0.500 |
| 4 | 0.952 | 0.395 |
| 5 | 0.244 | 0.287 |
| 6 | 0.281 | 0.419 |
| 7 | 0.638 | 0.484 |
| 8 | 0.954 | 0.491 |
| 9 | 1.220 | 0.456 |
| 10 | 0.447 | 0.270 |
| 11 | 0.323 | 0.435 |
| 12 | 0.638 | 0.495 |
| 13 | 0.862 | 0.458 |
| 14 | 0.911 | 0.395 |
| 15 | 1.308 | 0.495 |
| 16 | 0.394 | 0.290 |
| 17 | 0.292 | 0.395 |
| 18 | 0.638 | 0.493 |
| 19 | 0.770 | 0.468 |
| 20 | 0.862 | 0.470 |
| 21 | 0.888 | 0.463 |
| 22 | 0.862 | 0.470 |
| 23 | 0.770 | 0.468 |
| 24 | 0.638 | 0.493 |
| 25 | 0.292 | 0.395 |
| 26 | 0.394 | 0.290 |
| 27 | 1.308 | 0.495 |
| 28 | 0.911 | 0.395 |
| 29 | 0.862 | 0.458 |
| 30 | 0.638 | 0.495 |
| 31 | 0.323 | 0.435 |
| 32 | 0.447 | 0.270 |
| 33 | 1.220 | 0.456 |
| 34 | 0.954 | 0.491 |
| 35 | 0.638 | 0.484 |
| 36 | 0.281 | 0.419 |
| 37 | 0.244 | 0.287 |
| 38 | 0.952 | 0.395 |
| 39 | 0.852 | 0.500 |
| 40 | 0.504 | 0.439 |
| 41 | 0.100 | 0.100 |

**5. Chirped IDT properties**

In this work, to test the broadband performance of metasurfaces, a broadband excitation method is required. The chirped IDT provides an excellent solution through its gradient electrode width and spacing distribution which is based on the working frequency. Our IDT consists of a total of 74 electrode pairs with an aperture of 63.5 µm. In Fig. S4a, the variation of pitch and metallization ratio with electrode pair indexing is presented, exhibiting a linear trend. In Fig. S4b, we present the experimentally measured values of |S11|, which characterize the intensity of the reflection of the input electrical signal. A smaller value of |S11| indicates a greater conversion of electrical energy into mechanical energy (SAW), corresponding to a higher level of excitation. A value of |S11| equal to 0 indicates that the electrical signal is fully reflected, and no surface acoustic wave is excited. The results in Fig. S4b demonstrate the broadband operating performance of the IDT, especially from 550MHz to 650 MHz. For this work, we only need to excite SAW at the appropriate frequency, without requiring particularly high intensity, as long as they are strong enough to be detected by optical probe. Therefore, impedance optimization for higher intensity excitation was not performed.


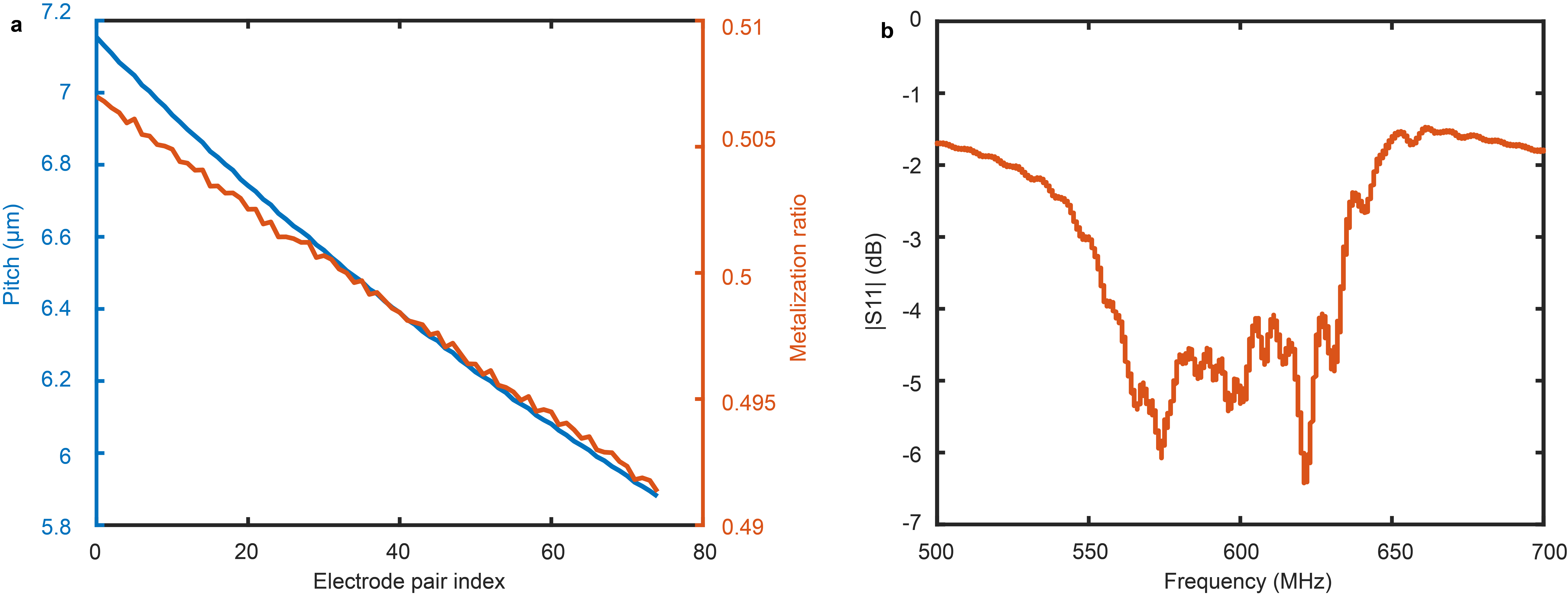


Fig.S4 (a) Variation of pitch (blue curve) and metallization ratio (red curve) with index of electrode pair; (b) Measured |S11| response of our chirped IDT.

**6. Optical characterization**.

The 25 µm × 25 µm scanning area is shown in Fig.S5a. An analog signal generator, Agilent N5181A, was employed to drive the interdigital transducer at a single frequency for each test, with the drive power consistently set at 15 dBm. Out-of-plane displacement fields were measured using a laser scanning heterodyne interferometer, with measurement steps of 0.25 µm (550 MHz, 560 MHz) or 0.3 µm (from 570 MHz to 650 MHz). As shown in Fig.S5b, laser beam from frequency stabilized HeNe (*λ* = 633 nm) source (Thorlabs HRS015B) is split into two frequency-shifted paths using an acousto-optic modulator driven at 110 MHz (AA Opto Electronic MTS110-A3-VIS). The first frequency-shifted beam served as a reference and was sent to a polarizing beam splitter. In contrast, the second beam propagated through a half-wave plate, the same polarizing beam splitter, a quarter-wave plate, and finally reached the substrate surface. A 0.6 µm spot focusing of the laser beam was achieved using a microscope objective (Olympus LMPLFLN100x). Upon reaching the sample, the laser beam returned, passing through the quarter-wave plate for a second time to rotate its polarization, which makes this beam to be reflected by the beam splitter and recombined with first beam. The combined beams were then directed to a fast photodiode (Alphalas UPD-200-SP) after passing through a linear polarizer. Finally, the electric signal captured by the photodiode was routed to an electrical spectrum analyzer (Anritsu MS2830A) for amplitude measurements or to an oscilloscope (Agilent DSO9254A) for phase measurements.


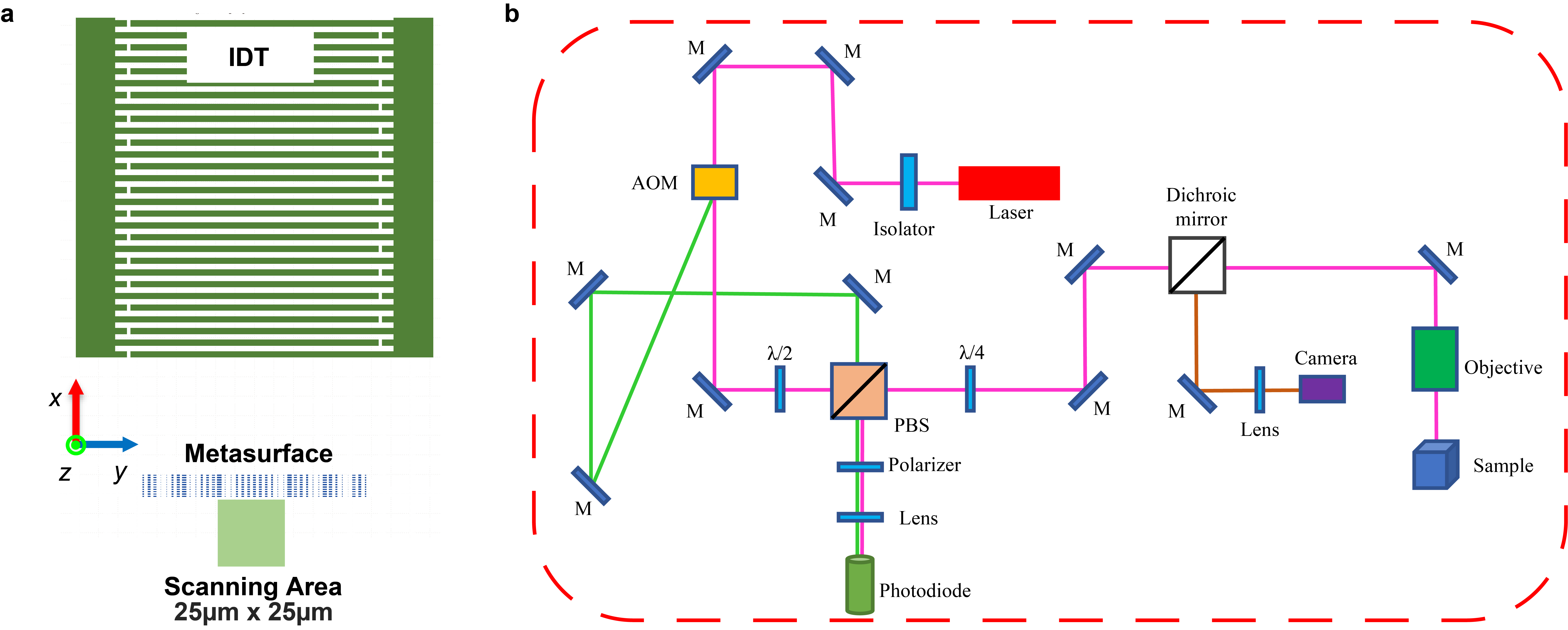


Fig. S5 Demonstration of (**a**) scanning area and (**b)** optical probe.
